# Cyclopia: The Face Predicts the Future

**DOI:** 10.7759/cureus.17114

**Published:** 2021-08-11

**Authors:** Michail Matalliotakis, Alexandra Trivli, Charoula Matalliotaki, Angelos Moschovakis, Eleftheria Hatzidaki

**Affiliations:** 1 Obstetrics and Gynecology, Venizeleio General Hospital, Heraklion, GRC; 2 Ophthalmology, Agios Nikolaos General Hospital, Agios Nikolaos, GRC; 3 Medicine, European University of Cyprus, Nicosia, CYP; 4 Neonatology and NICU, University Hospital of Heraklion, Heraklion, GRC

**Keywords:** cyclopia, etiopathogenesis, single eye, characteristics, risk of recurrence

## Abstract

The most extreme form of holoprosencephaly (HPE) is cyclopia and appears with a single characteristic midline diamond-shaped orbital structure and various facial, brain, and extrafacial features. We aimed to report a case of a cyclopic fetus diagnosed at the 22 weeks of the gestational age and further we reviewed the recent literature in order to highlight the etiopathogenesis and set goals for approaching such future pregnancies. Following the first-trimester assessment, in a 27-year-old pregnant woman, who underwent in vitro fertilization, the pregnancy was associated with a low risk for aneuploidies and a high risk for pre-eclampsia. On the anomaly scan, due to severe fetal brain maldevelopment and microcephale, HPE was suspected. Furthermore, three-dimensional ultrasound confirmed a common orbit in the midline of the face. Although the parents did not opt for amniocentesis and further postnatal management, parental karyotyping test did not detect any pathology. The pregnancy was terminated and the macroscopic examination of the aborted specimen revealed cyclopia, synophalmia, fussed eyelids with a proboscis on the upper midline of the face, and a malpositioned left ear. To conclude, cyclopia is not widely manifested, and different cyclopian disorders could still occur. Although this rare congenital abnormality is incompatible with life, the awareness of the spectrum of sonographic features and the appropriate genetic counseling can determine the outcome of current and forthcoming pregnancies.

## Introduction

Cyclopia represents an extremely rare congenital anomaly, with a frequency of 1/100,000 live and stillbirths. There is a predominance of female gender, possibly explained by the increased number of male miscarriages. Embryologically, it is characterized by the failure of the eye field to develop, resulting in a single socket in the place physiologically occupied by the base of the nose [1]. According to the Greek mythology, the first literature report is between eighth and seventh century BC in Homer’s Odyssey, where Polyphemus is the one-eyed character who threatens Odysseus [2]. As a part of holoprosencephaly (HPE), cyclopia is a malformation incompatible with life, whereas incomplete cleavage or absence of prosenchephalon happens between 18th and 28th day of embryonic period, thus affecting both the face and brain. According to the De Meyer’s classification, three forms of increasing severity of HPE exist: lobar, semilobar, and alobar [3]. Cyclopia presents mostly as the alobar type, where there is complete or near-complete deficiency of interhemispheric fissure and varying degrees of separation of the prosenchephalon [1,3]. Of note, in a recent study, El-Dessouky et al. investigated the craniofacial, extrafacial, and abnormal karyotyping of 25 fetuses with HPE. They confirmed seven cases with cyclopia (28%) in which all of them had alobar subtype [3]. According to the literature, various risk factors have been implicated for the pathogenesis of cyclopia with sporadic cases and Patau syndrome (trisomy 13) being the most common [4]. The diagnosis of cyclopia is mostly established after 20 weeks of gestation by ultrasonography (USG) [5]. In the framework of the present work, we aimed to analyze a rare case of a cyclopic fetus diagnosed on the two-dimensional (2D) and three-dimensional (3D) USG. Furthermore, we aimed to evaluate the recent literature in order to provide future trends in following such pregnancies.

## Case presentation

**Figure 1:**
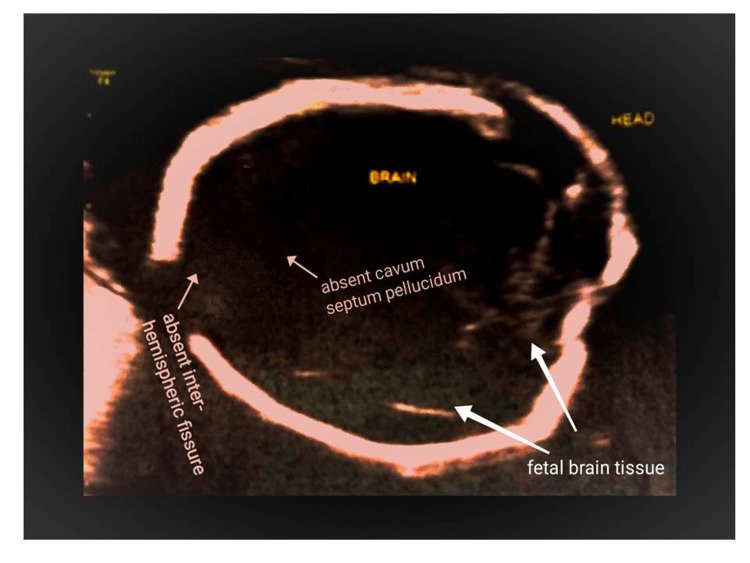
Abnormal fetal brain development

**Figure 2:**
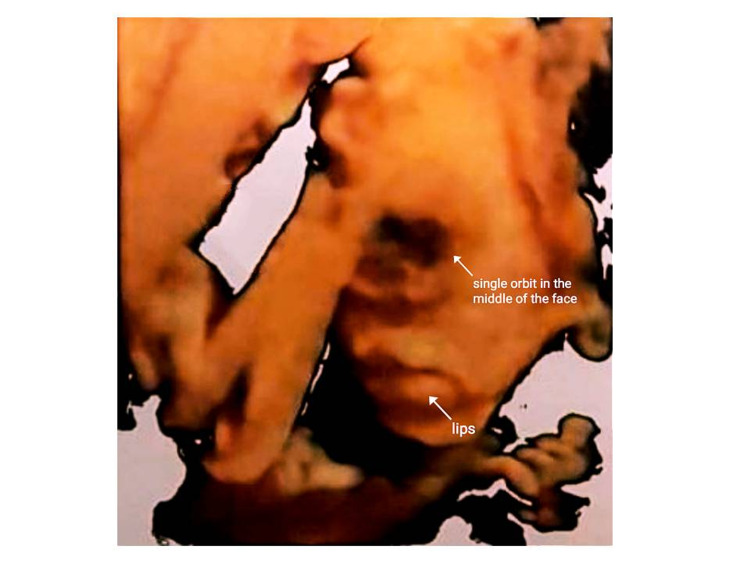
Three-dimensional reconstruction of the fetal face revealing the cyclopia, at 22 weeks of the gestational age

**Figure 3:**
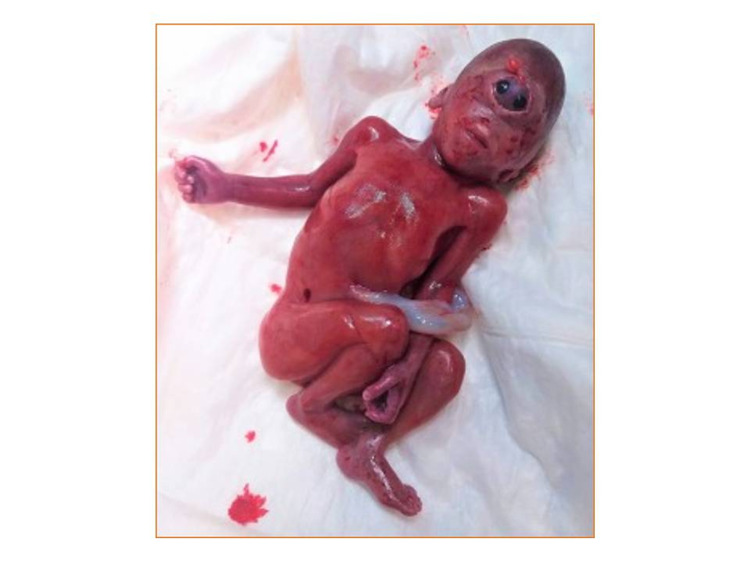
Delivered cyclopic fetus. Synophthalmia with a small proboscis at the supraorbital midline angle of the diamond-shaped eye structure and a prominent malpositioned left ear are confirmed

A 27-year-old, primigravida underwent in vitro fertilization (IVF) due to unilateral blocked fallopian tube and oligospermia of her partner. She reported no significant medical and family history, except for hypothyroidism and a uterine fibroid of no clinical importance. Following the first-trimester assessment, the pregnancy was associated with an increased risk of pre-eclampsia and a low risk for aneuploidies. At 13 weeks of gestational age, due to increased risk for pre-eclampsia a daily dose of 160 mg of aspirin was prescribed. Subsequently the woman presented for the second-trimester screening for fetal defects at 22 weeks. On the scan, evaluation of the fetal anatomy showed abnormal development of the brain with a suspicion of alobar HPE (Figure 1). Furthermore, microcephaly was diagnosed due to a prominent decrease of head circumference (53.2 mm) on the scan. A common orbit in the upper half of the face was confirmed by 3D USG; thus, the patient was referred to the fetal medicine unit for further management (Figure 2). Genetic counseling was performed. Parental karyotyping test did not detect any pathology. Moreover, amniocentesis and fetal magnetic resonance imaging (MRI) were offered but the couple did not opt for the procedures. Furthermore, the TORCH (toxoplasmosis, other, rubella, cytomegalovirus, herpesvirus) serum tests did not reveal any abnormality. With the informed consent of the patient, the pregnancy was terminated by mifepristone and prostaglandin induction after proper counseling. A female aborted fetus was delivered with a weight of 350 grams. At gross examination, we confirmed cyclopia, synophalmia, fussed eyelids with a small proboscis on the midline of the face, and a malpositioned left ear (Figure 3). On detailed macroscopic examination, no additional structural abnormalities were detected.

 

## Discussion

**Figure 4:**
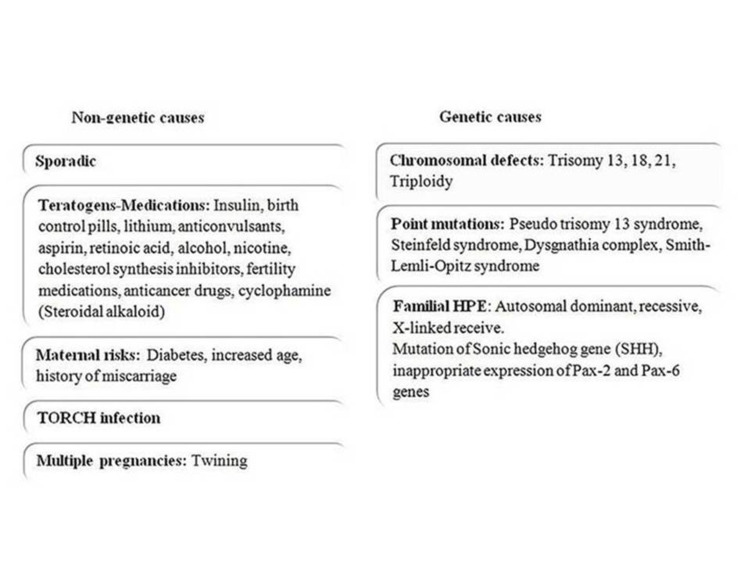
Summary of the risk factors related to cyclopia and holoprosencephaly

In the present study, we report a fetus with cyclopia at 22 weeks of gestational age. Two-dimensional transabdominal USG confirmed HPE, while 3D transabdominal USG demonstrated a single orbit in the middle of the face with a proboscis above the eye. According to Sedano and Gorling, various theories explain a possible pathogenesis of this developmental abnormality. One hypothesis reveals that the anomalous differentiation of the prechordal mesoderm in the central part of the head results in the irregular growth of the frontonasal process, which leads to the absence of facial structures such as the nose, the philtrum, and ethmoidal and premaxillary bones [6]. This phenomenon drives to the abnormal position of the eyes in the midline. Another theory supports that brain malformation is the primary cause and that ocular anomalies develop from deficient division of a single primordium. Additionally, the arterial circulation theory postulates that the aortic arch plexuses undergo fusion in the midline, and thus a mechanical traction on the growing optical primordium tends to fuse the optical anlagen in the midline [6]. Well of note, heterogeneous risk factors have been involved as possible causes. In Figure 4, we summarize the major risk factors as reported by the recent literature [1,2,4,7]. In our case, even though the couple did not opt for amniocentesis, IVF procedure presets a possible risk factor, but not aspirin administration, since it was administered after the embryonic period.

Noteworthy, Orioli et al. conducted an epidemiological study in a large data set including 257 infants with cyclopia. They described associated fetal malformations and maternal characteristics among pregnancies with cyclopia. They confirmed a high proportion of cases with chromosomal abnormalities (31%) and cases not typically related to HPE (31%). On the other hand, they did not observe a correlation with increased maternal age and twining [1]. Although the uniqueness of this pathology does not allow epidemiological studies to clearly demonstrate the etiopathogenesis of this entity, recently, Kamaldeep Singh et al. revised the literature regarding the cause, development, and diagnosis of cyclopia. In that study, they included several case reports related to cyclopia and highlighted the patient’s medical history, the clinical characteristics of the mothers and fetuses, as well as the pregnancy outcomes [4]. The sonographic diagnosis is often established in the late first and second trimesters based on the intracranial findings and facial abnormalities. In parallel, fetal MRI allows us to evaluate fetal brain malformations in detail [5,8-10]. Facial abnormalities demonstrate the most characteristic and severe manifestations of cyclopic fetuses. As previously described, cyclopia refers to a single midline diamond-shaped orbit that contains a single eye, or partially fused eyes (synophthalmia), or absence of the orbital structures (anophalmia) with maldeveloped eyelids. Furthermore, various studies reported a single optic trunk, a hypoplastic or absent optic nerve, incomplete separation of ocular muscles and possible clefts of the external ocular angles, and lid colobomas. In our case, two fused eyes in a single eye socket and fused eyelids were confirmed. Of note, the nose typically appears with a form of proboscis in the upper or lower angle of the diamond-shaped orbital cavity, which microscopically contains respiratory epithelium with mucus glands, cartilage, and bone. Other specimens have a lack of proboscis. In the present study, a small skin covering the proboscis above the fused eyes was confirmed. As far as the oral manifestations are concerned, several malformations have been observed such as a triangular-shaped mouth due to abnormal upper lip, microstomia or astomia, cleft lip or palate, and maldevelopment of parotid gland. The ears can be partially or totally absent or malpositioned. In our report, the left ear was misplaced closer to the mouth [6,11,12]. Worthy of note, most of the cases are stillborn, but there are full-term infants that have survived for few minutes to hours. The coexistence of extrafacial abnormalities such as polydactyle, omphalocele, genital, renal, and visceral abnormalities almost always associates with a stillbirth. As far as the differential diagnosis is concerned, asymmetrical monophalmia, cryptophthalmos, and otecephaly are predominated [2,5,6]. In term of management, termination of the pregnancy should be offered in all cases after a detailed prenatal examination and appropriate genetic counseling. Postnatal chromosomal analysis and gross examination of the specimen can further contribute to the diagnosis of cyclopia. Genetic counseling is important in order to evaluate the risk of recurrence. These are reported as 6% in chromosomally normal fetuses, 1% in an abnormal karyotype, and 50% or 25% in autosomal-dominant and -recessive traits, respectively [9].

## Conclusions

We report an extremely rare case of HPE that presented with cyclopia, facial dysmorphy, and a lower implanted left ear, with the purpose to highlight the etiopathogenesis, diagnosis, management, and, more importantly, prognosis of such a birth defect. With advances in diagnostic imaging, proper USG examination and genetic counseling are essential, since the face abnormalities can predict the risk of recurrence and pregnancy outcome. Collaboration between specialties as obstetrics, ophthalmology, and neonatology plays an important role in the approach of such cases.
